# Propolis supplementation on inflammatory and oxidative stress biomarkers in adults: a systematic review and meta-analysis of randomized controlled trials

**DOI:** 10.3389/fnut.2025.1542184

**Published:** 2025-05-12

**Authors:** Hossein Bahari, Mostafa Shahraki Jazinaki, Mohsen Aliakbarian, Mohammad Rashidmayvan, Haniyeh Golafrouz, Iman Rahnama, Rozita Khodashahi, Mahsa Malekahmadi

**Affiliations:** ^1^Transplant Research Center, Clinical Research Institute, Mashhad University of Medical Sciences, Mashhad, Iran; ^2^Student Research Committee, Mashhad University of Medical Sciences, Mashhad, Iran; ^3^Department of Nutrition, Food Sciences and Clinical Biochemistry, School of Medicine, Social Determinants of Health Research Center, Gonabad University of Medical Science, Gonabad, Iran; ^4^Rajaei Cardiovascular Medical and Research Center, Iran University of Medical Sciences, Tehran, Iran; ^5^Clinical Research Development Unit, Imam Reza Hospital, Faculty of Medicine, Mashhad University of Medical Sciences, Mashhad, Iran; ^6^Department of Infectious Diseases and Tropical Medicine, Faculty of Medicine, Mashhad University of Medical Sciences, Mashhad, Iran; ^7^Imam Khomeini Hospital Complex, Tehran University of Medical Sciences, Tehran, Iran

**Keywords:** propolis, oxidative stress, inflammation, systematic review, meta-analysis

## Abstract

**Background:**

Although a large number of trials have observed the anti-inflammatory properties of propolis, the currently available research remains controversial regarding its beneficial health effects. Hence, the purpose of this study was to examine the effect of propolis on inflammatory and oxidative stress markers in adults.

**Methods:**

A comprehensive search was performed in Scopus, Web of Science, and PubMed/Medline to find relevant randomized controlled trials (RCTs) until January 2024. The overall effect sizes were calculated using the random-effects model and expressed as weighted mean differences (WMD) with a 95% confidence interval (CI). The possible heterogeneity between included trials was assessed by performing Cochran’s Q test.

**Results:**

In total, 27 trials with 29 treatment arms were eligible for inclusion in this review. This meta-analysis revealed that propolis consumption led to a significant decrease in C-reactive protein (CRP) (WMD: –1.23; 95%CI: –1.76, –0.69; *p* < 0.001), Interleukin-6 (IL-6) (WMD: –1.52; 95%CI: –2.10, –0.93; *p* < 0.001), Tumor necrosis factor-α (WMD: –1.15; 95%CI: –1.75, –0.55; *p* < 0.001), and Monocyte chemoattractant protein-1 (MCP-1) (WMD: –35.33; 95%CI: –50.28, –20.37; *p* < 0.001), and a significant increase in total antioxidant capacity (TAC) (WMD: 0.32; 95%CI: 0.12, 0.51; *p* = 0.001), Glutathione (GSH) (WMD: 4.71; 95%CI: 3.17, 6.25; p < 0.001), and Glutathione peroxidase (GPx) (WMD: 44.75; 95%CI: 5.10, 84.40; *p* = 0.02). However, there were no significant effects on IL-10, IL-2, IL-8, pro-oxidant-antioxidant balance (PAB), malondialdehyde (MDA), and superoxide dismutase (SOD) in comparison to the control group.

**Conclusion:**

Propolis supplementation appears effective in reducing inflammation and oxidative stress by enhancing antioxidant capacity and reducing specific inflammatory markers. However, variations in study designs, dosages, and participant characteristics contribute to the heterogeneity of results. Further well-designed RCTs are needed to confirm these findings and determine the optimal dosage and long-term effects. Given its potential anti-inflammatory and antioxidant properties, propolis may serve as a complementary approach in managing inflammation-related conditions, though its clinical application requires further validation.

**Systematic review registration:**

https://clinicaltrials.gov/, identifier CRD42023474033.

## 1 Introduction

Honeybees (mostly Apis mellifera) create propolis, a natural resinous mixture, by combining exudate collected from various plant sources with salivary enzymes and wax (1). Bees use propolis to patch up damaged areas of their honeycombs, keeping the interior at a constant temperature and humidity while also creating a sterile space and guarding the entrance from potential predators. Traditional medicine has used propolis for a very long time because it has many health benefits (2). The chemical composition of propolis varies greatly depending on a number of factors, including the time of year, the type of vegetation at the collection site, and the species of bees involved. The active components of propolis have been identified in more than 300 samples from multiple regions of the world. These include phenolic acids and related esters, flavonoids, terpenes, aromatic aldehydes and alcohols, stilbenes, b-steroids, and fatty acids (1, 3, 4). Numerous chronic diseases have been found to be helped by propolis because of its antimicrobial, antiviral, antifungal, antiprotozoal, antioxidant, anti-inflammatory, immunomodulatory, antihyperglycemic, antihypertensive, antiproliferative, and hepatoprotective characteristics (1). The propolis used in previous studies has antioxidant and anti-inflammatory properties (5–7). In addition, a recent systematic review suggested that propolis could alleviate oxidative stress, renal damage, and inflammation status (8).

Propolis modulates the immune system by targeting both the innate and adaptive immune responses (9). This natural product can raise the levels of anti-inflammatory agents such as Interleukin-10 (IL-10) (10) and lower the levels of pro-inflammatory factors like Interferon (IFN-γ), IL-1β (11), Tumor necrosis factor-α (TNF-α), IL-6 (12), ICAM-1 (intercellular adhesion molecule), leukotrienes D4, and prostaglandins E2 and F2α (10). Recent scientific research has indicated that propolis may play a significant role in the treatment of inflammatory diseases (13) and immunological disorders (9). Earlier meta-analyses showed that propolis supplementation significantly decreased C-reactive protein (CRP), TNF-α, and IL-6 (12). Another key antioxidant component of propolis is caffeic acid phenethyl ester (CAPE), which works by blocking the production of reactive oxygen species (ROS) (14). Propolis may reduce oxidative stress and inflammation, according to several studies (15–17). Today, we know that propolis has a lot of flavonoids, which are plant-based chemicals that stop the production of nitric oxide (NO), IL-1, and IL-6 (18). Phenolic acids, which have been found in abundance in propolis, are an additional immunomodulatory substance. Their molecular activity decreases the levels of NO, cytokines, and neutrophils by scavenging free radicals and inhibiting the production of nitric oxide and inflammatory cytokines by macrophages and/or neutrophils (19).

With a focus on propolis and inflammation, some studies found evidence of a possible connection between propolis and inflammation and oxidative stress. Two systematic reviews have been completed on the effects of propolis on inflammation and oxidative stress, respectively (12, 20). Because of the inconsistent evidence, availability of new data, and limitations of previous reviews, we aimed to conduct a new systematic review and meta-analysis of available randomized controlled trials (RCT) to investigate the effects of propolis supplementation on inflammatory and oxidative stress markers in adults.

## 2 Materials and methods

The Preferred Reporting Items of Systematic Reviews and Meta-Analysis (PRISMA) framework was considered the foundation for every step of the planning and execution of this systematic review and meta-analysis (21). Also, in the PROSPERO database, this systematic review’s protocol is available with the registration ID: CRD42023474033.

### 2.1 Search strategy

To find relevant RCTs that examined the impact of propolis on oxidative stress and inflammatory markers, the ISI Web of Science, PubMed, and Scopus databases were comprehensively searched until January 2024. There were no time or language constraints on this search. The search strategy that was used in each database contains main keywords such as: (intervention OR “randomized clinical trial” OR RCT OR “randomized controlled trial” OR “clinical trial” OR “trial” OR blinded OR parallel OR “Cross-Over”) AND (“propolis”). Lastly, in order to prevent missing any eligible trials, the Google Scholar search engine was manually searched and the reference lists of relevant papers were carefully examined.

### 2.2 Eligibility criteria

Two authors (M.R. and H.B.) independently screened the trials that were found through primary searches using the inclusion criteria of the current study. The eligibility criteria were designed by applying the PICOS framework as follows: Participant: adults, Intervention: propolis consumption, Comparison: control group, Outcomes: oxidative stress, and inflammation markers, Study: randomized controlled trials (22). All included studies had to meet the following criteria: (a) human interventional studies, (b) RCTs design, (c) propolis consumption as an intervention, (d) reporting the changes in the levels of inflammatory and oxidative markers, and (e) Intervention on the adult population (≥ 18 years).

### 2.3 Exclusion criteria

Studies met the following criteria excluded from the present review: non-RCT studies, and observational research such as case-control, cohort, cross-sectional, etc. Furthermore, short communication, review articles, letters to the editor, studies conducted on people younger than 18 years, comminution therapy, duration of intervention less than 1 week, and lack of an appropriate control group were other exclusion criteria.

### 2.4 Data extraction

Relevant required data was independently extracted from included trials by two investigators (M.A. and H.G.). The extracted items include the name of the first author, publication year, region or country, sample size for each group, characteristics of participants [health status, mean age, gender, and mean body mass index (BMI)]. Type of control group, features of intervention with propolis (type, dosage, and duration), and mean changes and SD of each marker level changes (or the level of each marker in the first and the end of intervention).

### 2.5 Quality assessment

By applying the approach proposed by the Cochrane Collaboration, the general risk of bias for each included study was evaluated (23). Based on this tool’s framework, the risk of bias was assessed in the following seven domains: incomplete outcome data, blinding of outcome assessment, random sequence generation, selective reporting, allocation concealment, other biases, and blinding of participants and personnel. Each subclass’s risk of bias was categorized into three levels: high, unclear, and low. If the number of high-risk bias subclasses is more than two, the general risk of bias is considered high. If there are two subclasses, the general risk of bias is deemed moderate, and if there are fewer than two, the general risk of bias is regarded as low (24, 25).

### 2.6 Data synthesis and statistical analysis

All conducted analyses were executed by using version 17 of STATA software (Stata Corp., College Station, TX). Furthermore, *p*-values less than 0.05 were identified as statistically significant (two-tailed). In order to assess the influence of propolis intake on identified outcomes, the pooled effect sizes were calculated according to the random effect model based on the mean changes and SDs in both intervention and control groups. Also, The overall effect size was expressed as weighted mean differences (WMD) and 95% confidence interval (95% CI) (26). The mean changes in the case of non-reporting were directly estimated by subtracting the level of the markers at the beginning of the intervention from the end. SD was also determined by using the following formula: Change SD = square root [(SDbaseline)^2^ + (SDfinal)^2^-(2 × R × SDbaseline × SDfinal)] (27). Interquartile range (IQR), standard Error (SEs), and 95% confidence interval by applying the method of Hozo et al. convert to SDs (28). Cochran’s *Q*-test and the I-squared statistic (I^2^) were used to assess the heterogeneity among the included studies (29). P-value < 0.05 was considered as the significant between-studies heterogeneity. In addition, if a significant heterogeneity was detected, then based on the I^2^ statistics measure the interpretation of levels of heterogeneity among the pooled effect size was done as follows: 40% < I^2^ < 75% identified as moderate, and 75% < I^2^ as high heterogeneity among combined effect sizes.

Subgroup analysis was conducted to find the source of heterogeneity among included studies based on the following pre-defined criteria (30): gender (males, females, and both sexes), duration of propolis intake (< 12 and ≥ 12 weeks), propolis dosage (<1,000 and ≥ 1,000 mg/day), age of subjects (< 50 and > 50 years), participants’ health status (non-alcoholic fatty liver disease (NAFLD), type 2 diabetes mellitus (T2DM), other conditions, and healthy), and baseline BMI (normal, overweight, and obesity). Egger’s regression test and visual examination of funnel plots were used to assess publication bias for each outcome. (31). The impact of each of the effect sizes on the overall effect size was investigated for each outcome by conducting a sensitivity test with the leave-one-out approach (32). Polynomial modeling analyses and Meta-regression were performed to investigate the non-linear and linear relationship between the characteristics of propolis intervention (dose and duration) and changes in the levels of each of the oxidative and inflammatory stress markers, respectively (33).

### 2.7 GRADE analysis

In this meta-analysis, the GRADE (Grading of Recommendations Assessment, Development, and Evaluation) protocol was applied to assess the level of the evidence’s certainty (34). Based on this framework, the limitations of the evidence were evaluated in 5 sections: inconsistency, indirectness, publication bias, risk of bias, and imprecision.

Limitations in each domain were categorized into the following three classes: very serious limitations, serious limitations, and no serious limitations. Lastly, four degrees of evidence quality were identified: very high, high, moderate, and low.

## 3 Results

### 3.1 Study selection

**FIGURE 1:**
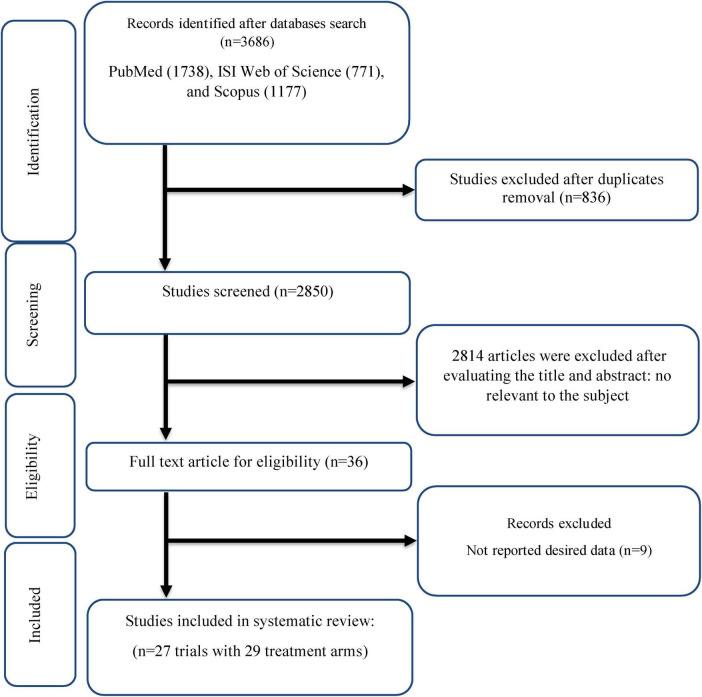
Flow chart of study selection for inclusion trials in the systematic review.

As shown in Figure 1, After 3,686 studies were obtained by initial search, 836 duplicate papers were removed. Among the remaining 2,850 studies that were screened, 2,814 did not meet the eligibility criteria for this review. The full text of 36 studies was evaluated, of which nine trials were excluded because they did not provide the necessary data. Finally, 27 studies (with 29 arm treatments) with 1,539 participants were included in this review (Figure 1) (6, 15, 17, 35–58).

### 3.2 Study characteristics

The included trials were published between 2003 and 2023. The study countries included Egypt (35, 43), Japan (37, 41, 55), China (6, 42, 46, 55), Iran (15, 17, 38, 40, 44, 47–53, 56–58) Chile (45), Brazil (36, 54), and Serbia (39). Among the included trials, 6 were conducted on females (15, 43, 50, 55–57) 2 on males (17, 48), and 20 on both sexes (6, 35–42, 44–47, 49–54, 58). The sample size of the treatment arms ranged from 24 (52) to 99 participants (49). The mean age of the participants varied from 24.2 (17) to 75 years (55). Also, the mean BMI varied from 21.07 (52), and 33.29 (58) kg/m^2^. The participants in the four included studies were healthy (6, 37, 45, 55). Also, in one included study, participants were patients with cardiovascular disease, diabetes mellitus, or overweight, or at least one altered parameter in the following markers: lipid profile, fasting glycemia, and blood pressure (45). Furthermore, the participants of the rest of the trials were conducted on individuals with Asthma (35), T2DM (40–42, 44, 46, 47, 49), Breast cancer (43, 50), Asthenozoospermia (48), HIV (36, 54), COPD (39), Primary pneumosepsis (52), chronic kidney disease (CKD) (53), NAFLD (38, 51), T2DM and dyslipidemia (56), polycystic ovary syndrome (PCOS) (57), Metabolic Syndrome (58), or Rheumatoid arthritis (15). The propolis received in the included studies was in the form of drops (45), syrup (52), sachets (35), and pills (tablets and capsules) (6, 15, 17, 36–44, 46–51, 53–58). The daily dosage of propolis ranged from 160 mg (lowest dosage) (39) to 1,500 mg (highest dosage) (40, 44, 47, 48, 51, 54). Also, the intervention duration in the eligible trials was between 1.4 (shortest duration) (52) to 96 weeks (longest duration) (6). The features of the eligible trials are provided in Table 1.

**TABLE 1:** Characteristic of included studies in meta-analysis.

Studies	Country	Study design	Participant	Sex	Sample size		Trial duration (week)	Means age		Means BMI		Intervention		
					**IG**	**CG**		**IG**	**CG**	**IG**	**CG**	**Type**	**Dose (mg/day)**	**Control group**
Khayyal et al. (35)	Egypt	Parallel, PC	Asthma	B	22	23	8	19-52	19-52	NR	NR	Propolis (aqueous extract)	260	Placebo
Fukuda et al. (41)	Japan	Parallel, R, PC, DB	T2DM	B	41	39	8	63.7	62.9	25	25	Brazilian green propolis	226.8	Placebo
Zhao et al. (42)	China	Parallel, R, C	T2DM	B	32	33	18	59.5	60.8	25.8	27.2	Brazilian green propolis	900	Control
Ebeid et al. (43)	Egypt	Parallel, C	Breast cancer + radiotherapy	F	45	45	3	53.72	53.72	NR	NR	Propolis + radiotherapy	1,200	Radiotherapy
Afsharpour et al. (44)	Iran	Parallel, R, PC, DB	T2DM	B	30	30	8	51.81	49.05	26.78	26.74	Propolis	1,500	Placebo
Mujica et al. (45)	Chile	Parallel, R, PC, DB	At least one of following altered parameters: Fasting glycemia, Lipid profile, Blood pressure or Diabetes mellitus	B	35	32	12	48	44.5	27.9	28.2	Propolis solution	30 drops	Peppermint + fernet + synthetic
Zhu et al. (6)	China	Parallel, R, PC, DB	Elderly living at high altitude	B	30	30	96	72.28	73.23	NR	NR	Propolis	830	Placebo
Gao et al. (46)	China	Parallel, R, C	T2DM	B	25	30	18	57.7	60.6	25.2	26.6	Chinese propolis	900	Control
Afsharpour et al. (47)	Iran	Parallel, R, PC, DB	T2DM	B	30	30	8	51.81	49.05	26.78	26.74	Propolis	1,500	Placebo
Gholaminejad et al. (48)	Iran	Parallel, R, PC, DB	Asthenozoospermic men	M	29	28	10	31.61	30	27.02	26.52	Propolis	1,500	Placebo
Zakerkish et al. (49)	Iran	Parallel, R, PC, DB	T2DM	B	50	44	12	55.4	54.86	30.04	29.02	Iranian propolis	1,000	Placebo
Darvishi et al. (50)	Iran	Parallel, R, PC, DB	Breast cancer + chemotherapy	F	26	24	12	49.3	44.36	27.9	27.63	Propolis	500	Placebo
Soleimani et al. (17)	Iran	Parallel, R, PC, TB	Military cadets	M	24	25	4	24.21	24.2	23.82	23.22	Propolis	900	Placebo
Conte et al. (36)	Brazil	Parallel, R, PC, DB	HIV	B	20	20	12	41.6	38.75	NR	NR	Propolis	500	Placebo
Asama et al. (37)	Japan	Parallel, R, PC, DB	Elderly	B	35	33	24	66.6	66.1	22.6	22.8	Propolis	350	Placebo
Soleimani et al. (38)	Iran	Parallel, R, PC, DB	NAFLD	B	27	27	12	42.56	41.85	29.55	28.41	Propolis	500	Placebo
Zuza et al. (39)	Serbia	Parallel, R, C	COPD	B	20	20	4	66.2	62.6	NR	NR	N-acetyl cycteine + propolis	160	N-acetyl cysteine
Afsharpour et al. (40)	Iran	Parallel, R, PC, DB	T2DM	B	30	30	8	51.81	49.05	26.78	26.74	Propolis	1,500	Placebo
Nikbaf-Shandiz et al. (51)	Iran	Parallel, R, PC, DB	NAFLD	B	23	21	8	38.52	40.14	33.36	33	Propolis + calorie-restricted diet	1,500	Placebo + calorie-restricted diet
Pahlavani et al. (52)	Iran	Parallel, R, PC	Primary pneumosepsis	B	12	13	1.4	57.92	60.92	21.11	21.03	Propolis + melatonin	1,000	Melatonin
Pahlavani et al. (52)	Iran	Parallel, R, PC	Primary pneumosepsis	B	12	12	1.4	58.21	58.38	22.52	22.76	Propolis	1,000	Placebo
Anvarifard et al. (53)	Iran	Parallel, R, PC, DB	CKD	B	17	18	12	58.06	60.5	29.66	28.53	Propolis + bee pollen + oat	250	Wheat starch + bee pollen + oat
Tasca et al. (54)	Brazil	Parallel, R, PC, DB	HIV	B	20	20	12	41.6	38.7	NR	NR	Brazilian green propolis	1,500	Placebo
Kanazashi et al. (55)	Japan	Parallel, R, PC, DB	Healthy postmenopausal women	F	25	28	12	75	75	24	23	Propolis	1,362	Placebo
Moayedi et al. (56)	Iran	Parallel, R, PC, SB	T2DM + dyslipidemia	F	15	15	8	52.53	53.67	NR	NR	Propolis	500	Placebo
Moayedi et al. (56)	Iran	Parallel, R, PC, SB	T2DM + dyslipidemia	F	15	15	8	54.07	51.67	NR	NR	Propolis + exercise	500	Exercise
Abbasi et al. (57)	Iran	Parallel, R, PC, TB	PCOS	F	28	29	12	18-45	18-45	28.35	26.16	Propolis	500	Placebo
Sajjadi et al. (58)	Iran	Parallel, R, PC, DB	Metabolic Syndrome	B	33	29	12	54.27	53.86	32.56	34.03	Propolis	500	Placebo
Maddahi et al. (15)	Iran	Parallel, R, PC, DB	Rheumatoid arthritis	F	23	22	12	46.56	47.9	27.89	26.84	Propolis	1,000	Placebo

IG, intervention group; CG, control group; TB, triple-blinded; DB, double-blinded; SB, single-blinded; PC, placebo-controlled; CO, controlled; R, randomized; BMI, body mass index; CKD, chronic kidney disease; COPD, Chronic obstructive pulmonary disease; HIV, human immunodeficiency virus; NAFLD, non-alcoholic fatty liver disease; T2DM, type 2 diabetes mellitus; PCOS, polycystic ovary syndrome; NR, not reported.

### 3.3 Quality assessment

Based on the risk of bias assessment performed using the approach proposed by Cochrane, the general risk of bias was identified as high for 1 (46), and moderate for 1 (35), eligible trial. At the same time, the rest of the included trials had a low general risk of bias. Table 2 provides the results of the risk of bias assessment in each domain.

**TABLE 2:** Risk of bias assessment.

Study	Random sequence generation	Allocation concealment	Selective reporting	Other sources of bias	Blinding (participants and personnel)	Blinding (outcome assessment)	Incomplete outcome data	General risk of bias
Khayyal et al. (35)	L	U	H	L	U	H	L	Moderate
Fukuda et al. (41)	L	L	H	L	L	U	L	Low
Zhao et al. (42)	L	U	L	U	U	U	L	Low
Ebeid et al. (43)	U	U	L	L	U	L	L	Low
Afsharpour et al. (44)	L	U	H	L	L	L	L	Low
Mujica et al. (45)	L	U	L	L	L	U	L	Low
Zhu et al. (6)	L	U	H	L	U	U	L	Low
Gao et al. (46)	H	U	L	L	H	U	H	High
Afsharpour et al. (47)	L	U	L	L	L	L	L	Low
Gholaminejad et al. (48)	L	L	L	L	L	U	L	Low
Zakerkish et al. (49)	L	L	L	L	U	L	L	Low
Darvishi et al. (50)	L	L	L	L	L	U	L	Low
Soleimani et al. (17)	L	L	H	L	L	L	L	Low
Conte et al. (36)	L	U	L	L	L	U	L	Low
Asama et al. (37)	L	U	H	L	L	U	L	Low
Soleimani et al. (38)	L	L	H	L	L	L	L	Low
Zuza et al. (39)	L	L	H	L	U	U	L	Low
Afsharpour et al. (40)	L	U	H	L	L	L	L	Low
Nikbaf-Shandiz et al. (51)	L	L	L	L	L	U	L	Low
Pahlavani et al. (52)	U	U	L	L	U	U	L	Low
Anvarifard et al. 2023 (53)	L	L	H	U	L	U	L	Low
Tasca et al. (54)	L	U	L	L	L	U	L	Low
Kanazashi et al. (55)	L	L	L	L	L	U	L	Low
Moayedi et al. (56)	L	L	H	U	L	U	L	Low
Abbasi et al. (57)	L	L	H	L	L	L	L	Low
Sajjadi et al. (58)	L	L	H	L	L	U	L	Low
Maddahi et al. (15)	L	L	L	L	L	U	L	Low

L, low risk of bias; H, high risk of bias; U, unclear risk of bias. General low risk of bias: < 2 high risk. General moderate risk of bias: = 2 high risk. General bad: > 2 high risk.

### 3.4 Meta-analysis

#### 3.4.1 Impact of supplementing with propolis on CRP levels

**FIGURE 2:**
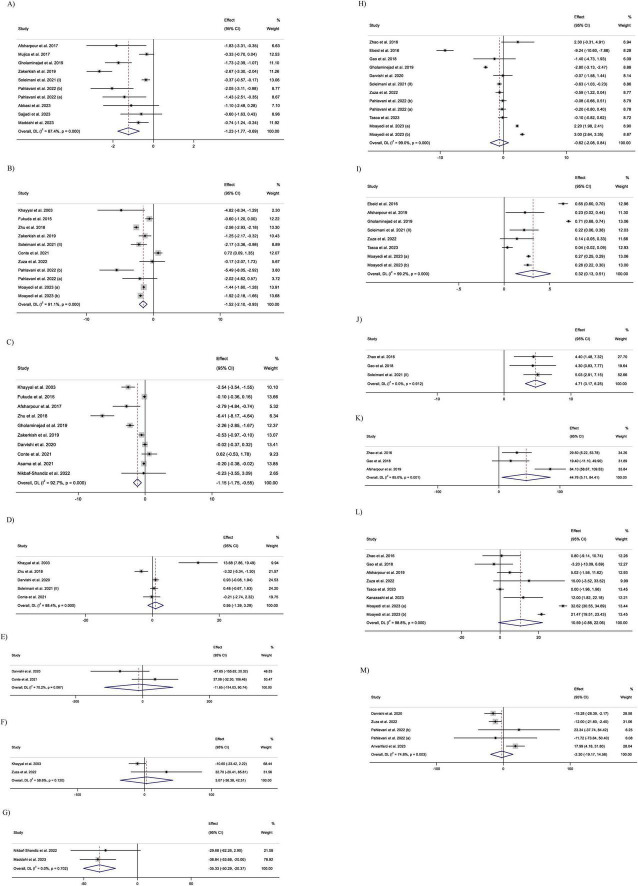
Forest plot detailing weighted mean difference and 95% confidence intervals (CIs) for the effect of Propolis intake on: **(A)** CRP (C-reactive protein, mg/L); **(B)** IL-6 (Interleukin-6, pg/mL); **(C)** TNF-α (Tumor necrosis factor-alpha, pg/mL); **(D)** IL-10 (Interleukin-10, pg/mL); **(E)** IL-2 (Interleukin-2, pg/mL); **(F)** IL-8 (Interleukin-8, pg/mL); **(G)** MCP-1 (Monocyte chemoattractant protein-1, pg/mL); **(H)** MDA (Malondialdehyde, nmol/mL); **(I)** TAC (Total Antioxidant Capacity, mmol/L); **(J)** GSH (Glutathione, μmol/L); **(K)** GPx (Glutathione Peroxidase, U/L); **(L)** SOD (Superoxide Dismutase, U/mL); and **(M)** PAB (Pro-oxidant-antioxidant balance).

Pooling 10 effect sizes demonstrated that propolis supplementation led to a significant decrease in the serum level of CRP (WMD: –1.23 mg/l; 95% CI –1.76 to –0.69; *p* < 0.001) (Figure 2A). However, significant heterogeneity was observed between the pooled trials (*p* < 0.001). Based on the I^2^ statistics measures (*I*^2^ = 87.4%) the heterogeneity among the included studies was identified as high (*I*^2^ > 75%). Subgroup analysis revealed that consumption of propolis in individuals with metabolic syndrome or obesity did not have a significant impact on the serum CRP levels (Table 3). Meta-regression demonstrated that the dose (coefficients = –316.08, P_linearity_ = 0.08; Supplementary Figure 1A) and duration (coefficients = 2.26, P_linearity_ = 0.23; Supplementary Figure 2A) of propolis supplementation were not sources of heterogeneity. Also, no significant linear relationship was detected between them and serum CRP changes.

**TABLE 3:** Subgroup analyses of Propolis consumption on inflammation and oxidative stress in adults.

	Number of effect sizes	WMD (95%CI)	*P*-value	heterogeneity		
				**P heterogeneity**	***I* ^2^**	**P between sub-groups**
**Propolis intake on serum CRP (mg/L)**						
Overall effect	10	–1.23 (–1.76, –0.69)	**<0.001**	<0.001	87.4%	
**Gender**						
Both	7	–1.26 (–1.96, –0.55)	**<0.001**	<0.001	90.1%	0.068
Female	2	–0.78 (–1.26, –0.31)	**0.001**	0.631	0.0%	
Male	1	–1.73 (–2.39, –1.07)	**<0.001**	–	–	
**Age**						
>50	5	–1.76 (–2.56, –0.96)	**<0.001**	0.014	68.2%	0.028
<50	5	–0.74 (–1.17, –0.30)	**0.001**	0.002	76.9%	
**Trial duration (week)**						
<12	4	–1.74 (–2.21, –1.27)	**<0.001**	0.886	0.0%	0.046
≥12	6	–0.94 (–1.57, –0.30)	**0.004**	< 0.001	89.8%	
**Intervention dose (mg/day)**						
<1,000	3	–0.39 (–0.59, –0.19)	**<0.001**	0.546	0.0%	0.001
≥1,000	6	–1.72 (–2.43, –1.01)	**<0.001**	0.001	78.5%	
**Baseline BMI (kg/m^2^)**						
Normal (18.5-24.9)	2	–1.74 (–2.50, –0.98)	**<0.001**	0.428	0.0%	0.098
Overweight (25-29.9)	6	–0.82 (–1.26, –0.38)	**<0.001**	0.001	75.5%	
Obese (> 30)	2	–1.67 (–3.70, 0.35)	0.105	0.001	91.2%	
**Health status**						
Metabolic syndrome	1	–0.60 (–1.62, 0.43)	0.253	–	–	0.001
NAFLD	1	–0.37 (–0.57, –0.16)	**<0.001**	–	–	
T2DM	2	–2.53 (–3.15, –1.91)	**<0.001**	0.305	5.0%	
Others	6	–1.14 (–1.74, –0.55)	**<0.001**	0.001	75.8%	
**Propolis intake on IL-6 (pg/mL)**						
Overall effect	11	–1.52 (–2.10, –0.93)	**<0.001**	<0.001	91.1%	
**Gender**						
Both	8	–1.66 (–2.92, –0.40)	**0.010**	<0.001	93.1%	0.740
Female	2	–1.67 (–2.13, –1.19)	**<0.001**	0.002	89.4%	
Male	1	–2.17 (–3.35, –0.98)	**<0.001**	–	–	
**Age**						
>50	8	–1.67 (–2.19, –1.15)	**<0.001**	< 0.001	87.6%	0.499
<50	2	–0.68 (–3.51, 2.15)	0.639	<0.001	94.4%	
**Trial duration (week)**						
<12	8	–1.64 (–2.17, –1.11)	**<0.001**	<0.001	79.8%	0.598
≥12	3	–1.03 (–3.22, 1.15)	0.355	<0.001	97.4%	
**Intervention dose (mg/day)**						
<1,000	8	–1.34 (–1.98, –0.70)	**<0.001**	<0.001	93.2%	0.291
≥1,000	3	–2.73 (–5.23, –0.23)	**0.032**	0.009	78.6%	
**Baseline BMI (kg/m^2^)**						
Normal (18.5-24.9)	4	–2.28 (–4.02, –0.54)	**0.010**	0.001	82.8%	0.303
Obese (> 30)	1	–1.24 (–2.16, –0.32)	**0.008**	–	–	
Propolis intake on serum CRP (mg/L)						
**Health status**						
T2DM	4	–1.38 (–1.83, –0.92)	**<0.001**	<0.001	84.5%	0.405
Others	7	–2.10 (–3.72, –0.47)	**0.011**	<0.001	93.4%	
**Propolis intake on TNF-a (pg/mL)**						
Overall effect	10	–1.15 (–1.75, –0.55)	**<0.001**	<0.001	92.7%	
**Gender**						
Both	8	–1.15 (–1.82, –0.48)	**0.001**	<0.001	91.0%	0.001
Female	1	–0.02 (–0.37, 0.32)	0.887	–	–	
Male	1	–2.26 (–2.84, –1.67)	**<0.001**	–	–	
**Age**						
>50	5	–1.14 (–1.87, –0.41)	**0.002**	<0.001	92.8%	0.494
<50	4	–0.55 (–2.08, 0.99)	0.486	<0.001	93.4%	
**Trial duration (week)**						
<12	5	–1.65 (–3.11, –0.18)	**0.028**	<0.001	93.7%	0.290
≥12	5	–0.76 (–1.50, –0.01)	**0.046**	<0.001	92.4%	
**Intervention dose (mg/day)**						
<1,000	6	–0.91 (–1.57, –0.25)	**0.007**	<0.001	93.1%	0.419
≥1,000	4	–1.53 (–2.87, –0.18)	**0.026**	<0.001	87.6%	
**Baseline BMI (kg/m^2^)**						
Normal (18.5-24.9)	2	–0.17 (–0.32, –0.02)	**0.028**	0.541	0.0%	0.110
Overweight (25-29.9)	3	–1.56 (–3.44, 0.31)	0.103	<0.001	95.6%	
Obese (> 30)	2	–0.53 (–0.96, –0.09)	**0.016**	0.858	0.0%	
**Health status**						
Breast cancer	1	–0.02 (–0.37, 0.32)	0.887	–	–	0.075
NAFLD	1	–0.23 (–3.54, 3.08)	0.892	–	–	
T2DM	3	–0.52 (–1.18, 0.14)	0.124	0.012	77.3%	
Others	5	–2.01 (–3.60, –0.42)	**0.013**	<0.001	96.3%	
**Propolis intake on IL-10 (pg/mL)**						
Overall effect	5	0.95 (–1.39, 3.28)	0.428	<0.001	88.4%	
**Propolis intake on IL-2 (pg/mL)**						
Overall effect	2	–11.64 (–114.03, 90.73)	0.824	0.067	70.2%	
**Propolis intake on IL-8 (pg/mL)**						
Overall effect	2	3.06 (–36.37, 42.51)	0.879	0.120	58.6%	
**Propolis intake on MCP-1 (pg/mL)**						
Overall effect	2	–35.33 (–50.28, –20.37)	**<0.001**	0.702	0.0%	
**Propolis intake on MDA (nmol/mL)**						
Overall effect	12	–0.62 (–2.08, 0.83)	0.403	<0.001	99.0%	
**Gender**						
Both	6	–0.20 (–0.55, 0.13)	0.235	0.334	12.7%	0.341
Female	4	–0.89 (–3.34, 1.54)	0.471	<0.001	99%	
**Propolis intake on serum CRP (mg/L)**						
Male	2	–1.71 (–3.84, 0.40)	0.113	<0.001	98.5%	
**Age**						
>50	8	–0.45 (–2.05, 1.14)	0.578	<0.001	98.4%	0.656
<50	4	–0.95 (–2.47, 0.56)	0.218	<0.001	96.8%	
**Trial duration (week)**						
<12	8	–0.98 (–2.78, 0.82)	0.288	<0.001	99.3%	0.308
≥12	4	0.06 (–0.79, 0.91)	0.891	0.290	19.9%	
**Intervention dose (mg/day)**						
<1,000	7	0.78 (–0.55, 2.13)	0.250	<0.001	97.7%	0.011
≥1,000	5	–2.39 (–4.43, –0.35)	**0.021**	<0.001	98.3%	
**Baseline BMI (kg/m^2^)**						
Normal (18.5-24.9)	3	–0.35 (–0.71, –0.00)	0.050	0.235	31%	0.823
Overweight (25-29.9)	4	–0.63 (–3.00, 1.73)	0.601	<0.001	88.6%	
**Health status**						
Breast cancer	2	–4.66 (–13.64, 4.32)	0.309	<0.001	98.7%	0.001
T2DM	4	2.36 (1.56, 3.15)	**<0.001**	< 0.001	84.4%	
Others	6	–0.75 (–1.82, 0.33)	0.174	<0.001	96.3%	
**Propolis intake on TAC (mmol/L)**						
Overall effect	8	0.32 (0.12, 0.51)	**0.001**	<0.001	99.2%	
**Gender**						
Both	3	0.07 (–0.01, 0.17)	0.114	0.257	26.3%	0.011
Female	3	0.39 (0.18, 0.60)	**<0.001**	<0.001	98.9%	
Male	2	0.47 (–0.01, 0.95)	0.054	<0.001	97.2%	
**Age**						
>50	5	0.32 (0.15, 0.49)	**<0.001**	<0.001	97.8%	0.998
<50	3	0.32 (–0.19, 0.84)	0.223	< 0.001	99.5%	
**Trial duration (week)**						
<12	7	0.36 (0.16, 0.56)	**<0.001**	<0.001	99.2%	0.002
≥12	1	0.03 (–0.02, 0.09)	0.237	–	–	
**Intervention dose (mg/day)**						
<1,000	4	0.26 (0.24, 0.28)	**<0.001**	0.537	0.0%	0.378
≥1,000	4	0.41 (0.08, 0.74)	**0.014**	<0.001	99.3%	
**Baseline BMI (kg/m^2^)**						
Normal (18.5-24.9)	1	0.22 (0.06, 0.37)	**0.007**	–	–	0.282
Overweight (25-29.9)	2	0.49 (0.02, 0.96)	**0.040**	0.001	91%	
**Health status**						
Breast cancer	1	0.65 (0.59, 0.70)	**<0.001**	–	–	0.001
T2DM	3	0.26 (0.24, 0.28)	**<0.001**	0.878	0.0%	
Others	4	0.28 (–0.16, 0.72)	0.218	<0.001	99.3%	
**Propolis intake on GSH (μmol/L)**						
Overall effect	3	4.71 (3.17, 6.25)	**<0.001**	0.912	0.0%	
Propolis intake on GPx (U/L)						
Overall effect	3	44.75 (5.10, 84.40)	**0.027**	0.001	85%	
**Propolis intake on SOD (U/mL)**						
Overall effect	8	10.58 (–0.93, 22.11)	0.072	<0.001	98.8%	
**Gender**						
Both	5	1.05 (–2.02, 4.13)	0.501	0.292	19.2%	< 0.001
Female	3	23.07 (13.43, 32.71)	**<0.001**	< 0.001	97%	
**Trial duration (week)**						
<12	4	19.44 (9.36, 29.52)	**<0.001**	<0.001	97%	0.002
≥12	4	1.57 (–3.43, 6.57)	0.538	0.130	47%	
**Intervention dose (mg/day)**						
<1,000	5	14.44 (4.48, 24.40)	**0.004**	<0.001	96.6%	0.088
≥1,000	3	4.17 (–2.13, 10.49)	0.195	0.032	71%	
**Baseline BMI (kg/m^2^)**						
Normal (18.5-24.9)	1	12.00 (1.81, 22.18)	**0.021**	–	–	0.085
Overweight (25-29.9)	3	2.09 (–2.71, 6.89)	0.394	0.383	0.0%	
**Health status**						
Others	2	4.55 (–8.96, 18.07)	0.509	0.114	59.9%	0.623
T2DM	5	12.32 (2.00, 22.63)	**0.019**	<0.001	97.4%	
Healthy	1	12.00 (1.81, 22.18)	**0.021**	–	–	
**Propolis intake on PAB**						
Overall effect	5	–2.30 (–19.16, 14.56)	0.789	0.003	74.8%	

WMD, weighted mean differences; CI, confidence interval; BMI, body mass index; CRP, c-reactive protein; IL, interleukin; TNF-a, tumor necrosis factor-a; MCP-1, monocyte chemoattractant protein-1; MDA, malondialdehyde; TAC, total antioxidant capacity; SOD, superoxide dismutase; GSH, Glutathione; GPx, Glutathione Peroxidase; PAB, pro-oxidant antioxidant balance; NAFLD, non-alcoholic fatty liver disease; T2DM, type 2 diabetes mellitus.

#### 3.4.2 Impact of supplementing with propolis on IL-6 levels

After combining 11 effect sizes, a significant reduction in IL-6 serum levels was detected in the groups that received propolis (WMD: –1.52 pg/mL; 95% CI –2.10 to –0.93; *p* < 0.001) (Figure 2B). Furthermore, heterogeneity between the pooled trials was significant (; *p* < 0.001). I^2^ level (*I*^2^ = 91.1%), demonstrated a high heterogeneity among the combined effect sizes (*I*^2^ > 75%). Subgroup analysis demonstrated that propolis intake in trials conducted on participants aged less than 50 years, and in the studies with a duration of receiving propolis ≥ 12 weeks did not have a significant influence on the IL-6 serum levels (Table 3). Meta-regression revealed that the intervention features, including dosage and duration of propolis intake, were not sources of heterogeneity. Also, no significant linear relationship was identified between the characteristics of propolis intake and changes in IL-6 levels (dose: coefficients = -58.64, P _linearity_ = 0.32; Supplementary Figure 1B), and duration (coefficients = -0.61, P _linearity_ = 0.90; Supplementary Figure 2B).

#### 3.4.3 Impact of supplementing with propolis on TNF-α levels

Meta-analyzing 10 effect sizes demonstrated a significant decrease in serum TNF-α levels followed by propolis supplementation (WMD: –1.15 pg/mL; 95% CI –1.75 to –0.55; *P* < 0.001) (Figure 2C). However, a significant heterogeneity was observed between the included trials (*P* < 0.001). In addition, I^2^ statistics measures (*I*^2^ = 92.7%) indicated a high level of heterogeneity among pooled effect sizes (*I*^2^ > 75%). Subgroup analysis demonstrated a non-significant influence of propolis consumption on the TNF-a levels in the trials conducted on only females, subjects aged < 50 years, and participants with breast cancer, NAFLD, and type 2 diabetes (Table 3).

Meta-regression indicated that the dosage of propolis supplementation was not a source of heterogeneity, and there was no significant linear relationship between propolis dosage and changes in serum TNF-a levels (coefficients = -59.92, P _linearity_ = 0.50; Supplementary Figure 1C). However, the duration of intervention with propolis was identified as a source of heterogeneity. Also, a significant linear relationship was observed between the changes in the TNF-a level and duration of propolis supplementation (coefficients = –9.94, P _linearity_ = 0.009; Supplementary Figure 2C).

#### 3.4.4 Impact of supplementing with propolis on IL-10 levels

Meta-analyzing five effect sizes revealed that propolis supplementation had no significant impact on serum IL-10 levels (WMD: 0.95 pg/mL; 95% CI –1.39 to 3.28; *P* = 0.42) (Figure 2D). Also, a significant heterogeneity was identified between the pooled effect sizes (*P* < 0.001). I^2^ statistics (*I*^2^ = 85.9%) indicated a high level of heterogeneity among the included effect sizes (*I*^2^ > 75%).

#### 3.4.5 Impact of supplementing with propolis on IL-2 levels

Pooling of two effect sizes showed the non-significant influence of the propolis intake on IL-2 serum levels (WMD: –11.64 pg/mL; 95% CI (–114.03 to 90.73; *P* = 0.82) (Figure 2E). However, no significant heterogeneity was observed between the pooled effect sizes (*I*^2^ = 70.2%; *P* = 0.06).

#### 3.4.6 Impact of supplementing with propolis on IL-8 levels

Combining two effect sizes mentioned that propolis consumption had no significant impact on IL-8 serum levels [WMD: 3.06 pg/mL; 95% CI (–36.37 to 42.51; *P* = 0.87)] (Figure 2F). Furthermore, no significant heterogeneity was detected between the included trials (*I*^2^ = 58.6%; *P* = 0.12).

#### 3.4.7 Impact of supplementing with propolis on Monocyte chemoattractant protein-1 levels

Meta-analyzing two effect sizes revealed that propolis intake had a significant lowering effect on MCP-1 serum levels (WMD: –35.33 pg/mL; 95% CI (–50.28 to –20.37; *P* < 0.001) (Figure 2G). At the same time, no significant heterogeneity was detected between the pooled trials (*I*^2^ = 0.0%; *P* = 0.70).

#### 3.4.8 Impact of supplementing with propolis on Malondialdehyde levels

Meta-analyzing of 12 effect sizes demonstrated that propolis intake had no significant influence on MDA levels (WMD: –0.62 nmol/mL; 95% CI –2.08 to 0.83; *P* = 0.40) (Figure 2H). Furthermore, a significant heterogeneity was detected between the included effect sizes (*P* < 0.001). *I*^2^-values (*I*^2^ = 99.0%), indicated a high level of heterogeneity among the pooled effect sizes (*I*^2^ > 75%). Subgroup analysis indicated that consuming propolis with a dose of ≥ 1,000 mg/day and in the trials conducted on participants with type 2 diabetes led to a significant decrease in MDA levels (Table 3). Meta-regression showed that the dose (coefficients = –57.17, P_linearity_ = 0.15; Supplementary Figure 1E) and duration (coefficients = 0.52, P linearity = 0.37; Supplementary Figure 2E) of propolis supplementation were not sources of heterogeneity. Also, no significant linear relationship was observed between them and changes in MDA levels.

#### 3.4.9 Impact of supplementing with propolis on total antioxidant capacity (TAC) levels

Combining eight effect sizes revealed that propolis consumption significantly increased the TAC levels (WMD: 0.32 nmol/mL; 95% CI 0.12–0.51; *P* = 0.001) (Figure 2I). However, a significant heterogeneity was detected between included trials (*P* < 0.001). Based on the I^2^ measures (*I*^2^ = 99.2%), levels of heterogeneity among the pooled studies identified as high (*I*^2^ > 75%). Subgroup analysis demonstrated that propolis intake did not significantly change TAC levels in studies conducted only on males or on individuals aged < 50 years (Table 3). Meta-regression reported the absence of a significant linear relationship between the features (dose and duration) of the propolis intake and changes in TAC. It also showed that the dose (coefficients = 724.26, P _linearity_ = 0.43; Supplementary Figure 1F) and duration (coefficients = -2.33, P linearity = 0.67; Supplementary Figure 2F) of propolis supplementation were not sources of heterogeneity.

#### 3.4.10 Impact of supplementing with propolis on Glutathione (GSH) levels

Meta-analysis of three effect sizes demonstrated that propolis intake significantly increased the GSH levels (WMD: 4.71 μmol/L; 95% CI 3.17 to 6.25; *P* < 0.001) (Figure 2J). Also, there was no significant heterogeneity between the pooled effect sizes (*I*^2^ = 0.0%; *p* = 0.91).

#### 3.4.11 Impact of supplementing with propolis on Glutathione peroxidase (GPx) levels

**FIGURE 3:**
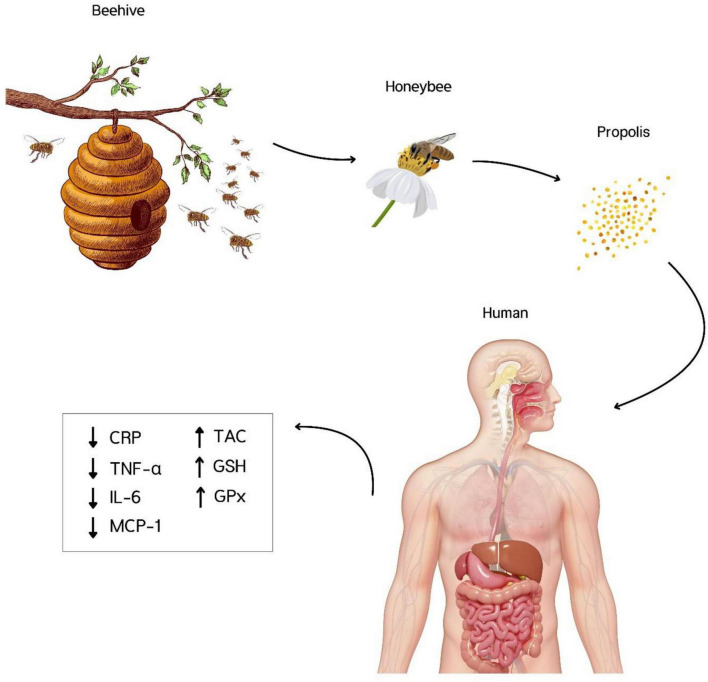
Propolis consumption significantly reduced C-reactive protein (CRP), Interleukin-6 (IL-6), Tumor necrosis factor-α (TNF-α), and Monocyte chemoattractant protein-1 (MCP-1), and increased total antioxidant capacity (TAC), Glutathione (GSH), and Glutathione peroxidase (GPx). There were no significant effects on IL-10, IL-2, IL-8, pro-oxidant-antioxidant balance, malondialdehyde, and superoxide dismutase.

Combining three effect sizes revealed that propolis consumption significantly increased GPx levels (WMD: 44.75 U/L; 95% CI 5.10–84.40; *P* = 0.02) (Figures 2K, 3). Also, a significant heterogeneity was observed between the pooled effect sizes (*P* = 0.001). Also, I^2^ levels (*I*^2^ = 85%) identified the levels of heterogeneity among the combined trials as high (*I*^2^ > 75%).

#### 3.4.12 Impact of supplementing with propolis on Superoxide dismutase (SOD) levels

Pooling eight effect sizes demonstrated that propolis intake did not significantly change SOD levels (WMD: 10.58 U/mL; 95% CI –0.93 to 22.11; *P* = 0.07) (Figure 2L). Also, a significant heterogeneity was mentioned between the included trials (*P* < 0.001). In addition, I^2^ measures (*I*^2^ = 98.8%) indicated a high heterogeneity among the pooled effect sizes (*I*^2^ > 75%). The subgroup analysis reported the significant enhancing effect of propolis consumption in studies conducted on only females, trials with an intervention duration of < 12 weeks or propolis supplemental dosage of <1,000 mg/day (Table 3). In addition, propolis intake in healthy individuals or patients with type 2 diabetes led to a significant increase in SOD levels.

Meta-regression reported a significant linear relationship between the duration of propolis intake and changes in SOD levels. Also, the duration of supplementation was identified as a source of heterogeneity (coefficients = -0.14, P _linearity_ = 0.01; Supplementary Figure 2G). However, the dosage of propolis supplementation was not the source of heterogeneity (coefficients = –24.14, P_linearity_ = 0.12; Supplementary Figure 1G). Furthermore, no significant linear relationship was detected between supplementation dosage and SOD level changes.

#### 3.4.13 Impact of supplementing with propolis on Pro-oxidant-antioxidant balance (PAB) levels

Combining five effect sizes demonstrated that propolis consumption had no significant impacts on PAB levels (WMD: –2.30 U/mL; 95% CI –19.16 to 14.56; *P* = 0.78) (Figure 2M). However, a significant heterogeneity was observed between the included effect sizes (*P* = 0.003). Based on the I^2^ levels (*I*^2^ = 74.8%), the heterogeneity among pooled trials was identified as moderate (40% < *I*^2^ < 75%).

### 3.5 Non-linear dose-response analysis

Fractional polynomial modeling demonstrated a significant non-linear relationship between propolis supplementation dosage and changes in SOD levels (coefficients = 14.77, P _*non–*linearity_ = 0.02). This analysis suggests that a daily supplement of 500 mg of propolis may induce a more pronounced increase in SOD levels compared to other dosages reported in the trials. Furthermore, it showed a significant non-linear relationship between the duration of propolis supplementation and changes in TNF-a (coefficients = –29.39, P _*non–*linearity_ = 0.01), MDA (coefficients = 10.05, P _*non–*linearity_ = 0.01), and SOD levels (coefficients = 39.18, P _*non–*linearity_ = 0.02).

It seemed that the duration of 8 weeks is an optimum duration for propolis intake to increase SOD compared to other duration of included trial duration. However, no significant non-linear relationship between the features (dose and duration) of propolis intake and changes in the levels of other outcomes was found (Supplementary Figures 3A-P).

### 3.6 Sensitivity analysis

The sensitivity analysis, which was performed to investigate the effect of the quality of each of the included studies on the overall effect size of each of the outcomes, reported that the impact of propolis supplementation on MCP-1 levels after removing the study conducted by Maddahi et al. (WMD: –29.68 pg/mL 95%CI: –62.25, 2.89), and for GPx after excluding Zhao et al. (WMD: 52.32 U/L 95%CI: –11.07, 115.71) changed significantly. Furthermore, omitting the Gao et al. (WMD: 12.51 U/mL 95%CI: 0.22, 24.81), or Tasca et al. (WMD: 12.55 U/mL 95%CI: 3.63, 21.47) led to a significant change in finding regarding the impact of propolis consumption on SOD levels. However, the pooled effect sizes of CRP, IL-6, TNF-a, IL-10, IL-2, IL-8, MDA, TAC, GSH, and PAB were not significantly affected by the presence of an effect size among pooled items.

### 3.7 Publication bias

The visual interpretation of funnel plots and the implementation of Egger regression and Begg rank correlation analyses showed that there was no significant publication bias among the evidence investigating the impact of propolis supplementation on any of the outcomes, including CRP (p_Begg_ = 0.28), IL-6 (p_Begg_ = 0.64), TNF-a (p_Begg_ = 0.07), IL-10 (p_Egger_ = 0.65), MDA (p_Begg_ = 0.73), TAC (p_Egger_ = 0.71), GSH (p_Egger_ = 0.29), GPx (p_Egger_ = 0.75), SOD (p_Egger_ = 0.64), and PAB (p_Egger_ = 0.66) (Supplementary Figures 4A-M).

### 3.8 GRADE analysis

The quality of evidence investigating the impact of propolis intake on MCP-1 or GSH was upgraded to very high due to a lack of serious limitations in none of the GRADE domains. The quality of evidence was considered moderate for CRP, IL-6, TNF-a, TAC, and GPx due to very serious inconsistency. Also, due to serious imprecision and serious inconsistency the quality of evidence for IL-2, IL-8, and PAB was identified as moderate, too. However, the certainty of evidence was downgraded to low quality for IL-10, SOD, and MDA due to serious imprecision and very serious inconsistency. The GRADE profile is shown in Table 4.

**TABLE 4:** GRADE profile of Propolis consumption for inflammation and oxidative stress in adults.

Outcomes	Risk of bias	Inconsistency	Indirectness	Imprecision	Publication Bias	Quality of evidence
CRP	No serious limitations	Very serious limitations^1^	No serious limitations	No serious limitations	No serious limitations	⊕⊕○○ Moderate
IL-6	No serious limitations	Very serious limitations	No serious limitations	No serious limitations	No serious limitations	⊕⊕○○ Moderate
TNF-a	No serious limitations	Very serious limitations	No serious limitations	No serious limitations	No serious limitations	⊕⊕○○ Moderate
IL-10	No serious limitations	Very serious limitations	No serious limitations	Serious limitations^3^	No serious limitations	⊕○○○ Low
IL-2	No serious limitations	Serious limitations^2^	No serious limitations	Serious limitations	No serious limitations	⊕⊕○○ Moderate
IL-8	No serious limitations	Serious limitations	No serious limitations	Serious limitations	No serious limitations	⊕⊕○○ Moderate
MCP-1	No serious limitations	No serious limitations	No serious limitations	No serious limitations	No serious limitations	⊕⊕⊕⊕ Very high
MDA	No serious limitations	Very serious limitations	No serious limitations	Serious limitations	No serious limitations	⊕○○○ Low
TAC	No serious limitations	Very serious limitations	No serious limitations	No serious limitations	No serious limitations	⊕⊕○○ Moderate
GSH	No serious limitations	No serious limitations	No serious limitations	No serious limitations	No serious limitations	⊕⊕⊕⊕ Very high
GPx	No serious limitations	Very serious limitations	No serious limitations	No serious limitations	No serious limitations	⊕⊕○○ Moderate
SOD	No serious limitations	Very serious limitations	No serious limitations	Serious limitations	No serious limitations	⊕○○○ Low
PAB	No serious limitations	Serious limitations	No serious limitations	Serious limitations	No serious limitations	⊕⊕○○ Moderate

CRP, c-reactive protein; IL, interleukin; TNF-a, tumor necrosis factor-a; MCP-1, monocyte chemoattractant protein-1; MDA, malondialdehyde; TAC, total antioxidant capacity; SOD, superoxide dismutase; GSH, Glutathione; GPx, Glutathione Peroxidase; PAB, pro-oxidant antioxidant balance.

^1^There is very high heterogeneity (*I*^2^ > 75%).

^2^There is high heterogeneity (*I*^2^ > 40%).

^3^There is no significant effect of Propolis consumption.

## 4 Discussion

Oxidative stress is a key factor in promoting inflammation and contributing to the onset of chronic conditions like cardiovascular diseases, diabetes, neurodegenerative disorders, and cancer (59). The accumulation of ROS resulting from an imbalance between their production and neutralization via DNA damage, lipid peroxidation, and protein modifications leads to tissue damage, activation of pro-inflammatory signaling pathways, and direct cell damage (60). Therefore, strategies to reduce oxidative stress and maintain a balance between ROS and antioxidants may help prevent or manage chronic diseases.

This current systematic review and meta-analysis study reveals that propolis reduces inflammation through the reduction of inflammatory markers including, CRP, TNF-α, and IL-6 in the intervention of more than 12 weeks and at the age of more than 50 years. It also lowers oxidative stress by decreasing MDA levels in doses of more than 1,000 mg/day and increasing TAC, GSH, GPX, and SOD in healthy people, women, and diabetes, and in the intervention of less than 12 weeks and the dose of less than 1,000 mg/day. In addition, the dose-response analysis in the present study showed that the optimum dose and duration for increasing SOD is 500 mg/day and 8 weeks, respectively.

Propolis supplementation shows a dose-dependent effect, with higher doses generally providing more pronounced benefits, particularly in reducing oxidative stress and improving glycemic control (47, 61, 62). The optimal duration for supplementation varies, with effective outcomes observed from as short as 1 week to as long as 6 months, depending on the health condition and desired outcomes (47, 55, 63, 64). These findings suggest that both the dose and duration of propolis supplementation should be tailored to the specific health goals of the individual.

The findings of this study are consistent with the meta-analysis of Hallajzadeh et al. (20). The difference is that in the present study, more studies on inflammatory factors and oxidative stress have been meta-analyzed, and the subgroup analyses performed show significant changes in oxidative stress markers with propolis intervention. The most recent meta-analysis also shows that intervention with propolis increases TAC, GSH, GPX, and MDA decreases with a dose ≥ 1,000 mg/day (61).

Propolis contains a variety of bioactive components, such as flavonoids, phenolic acids, and terpenoids, which contribute to its antioxidant activity (59). These compounds scavenge free radicals and inhibit ROS production, thereby reducing oxidative stress. Propolis flavonoids, specifically quercetin, and kaempferol derivatives, can directly neutralize ROS and suppress oxidative stress-induced damage (16).

Propolis has been demonstrated to influence signaling pathways related to inflammation. For example, it can suppress the activation of nuclear factor-kappa B (NF-κB), a crucial regulator of inflammation which demonstrated reduced NF-κB activation and subsequent downregulation of pro-inflammatory cytokines (65).

Furthermore, propolis has been discovered to regulate inflammatory mediators like cyclooxygenase-2 (COX-2) and inducible nitric oxide synthase (iNOS) (16). These enzymes are involved in the production of inflammatory molecules. A type of flavonoid in propolis, caffeic acid phenethyl ester (CAPE) inhibits the release of arachidonic acid from the cell membrane and prevents gene expression of LOX and COX enzymes (66). The compound CAPE disrupts the interaction between the ligand, LPS, and the receptor complex, TLR4/MD2, leading to the inhibition of Toll-like receptor 4 (TLR4) activation. The binding of LPS to a hydrophobic pocket in MD2 initiates the assembly of a receptor multimer consisting of two TLR4/MD2/LPS complexes. This, in turn, recruits adaptor proteins and activates intracellular signaling pathways. Dysregulation of the TLR4 receptor has been implicated in chronic inflammatory diseases (67).

Furthermore, propolis may modulate intracellular signaling pathways related to oxidative stress and inflammation. Experimental evidence demonstrated that propolis activates the nuclear factor erythroid 2-related factor 2 (Nrf2) pathway (68). Nrf2 is a master regulator of antioxidant defence and can promote the expression of antioxidant enzymes and phase II detoxifying enzymes, counteracting oxidative stress and reducing inflammation (69).

Although propolis is generally considered safe for consumption, it’s important to note that individuals may have varying sensitivities or allergies to bee products. While specific clinical trial studies on propolis supplements’ side effects are scarce, a few potential side effects such as allergic reactions, contact dermatitis, and gastrointestinal upset have been mentioned in general research and anecdotal reports. It’s worth emphasizing that these side effects are generally infrequent and mild (70).

The ability of propolis to modulate inflammatory and oxidative stress markers positions it as a potentially valuable treatment option for a range of diseases beyond its traditional uses. However, further research and standardization are necessary to fully realize its therapeutic potential. As scientific understanding of propolis grows, it could become an integral part of treatment strategies for neurodegenerative diseases, cardiovascular conditions, metabolic disorders, autoimmune diseases, and even cancer.

Propolis has shown promise in managing metabolic syndrome (MetS) and its associated chronic diseases, which are significant contributors to global mortality. Its antioxidant and anti-inflammatory properties help ameliorate symptoms by inhibiting advanced glycation end products (AGEs) and their receptors (RAGEs), as well as pro-inflammatory signaling cascades (71). In patients with type 2 diabetes mellitus (T2DM) and chronic periodontitis, propolis supplementation has been found to improve glycemic control and periodontal health. A clinical trial demonstrated significant reductions in hemoglobin A1c, fasting plasma glucose, and serum Nε-(carboxymethyl) lysine levels, alongside improved periodontal parameters (63). Propolis has been evaluated for its effects on non-alcoholic fatty liver disease (NAFLD), showing protective effects against hepatic steatosis and fibrosis. It significantly reduced liver stiffness and high-sensitivity C-reactive protein levels, indicating its potential as a therapeutic agent for NAFLD (38). Propolis may also benefit patients with rheumatoid arthritis (RA) by reducing inflammation and oxidative stress. It inhibits inflammatory pathways and reduces reactive oxygen species, potentially alleviating pain and improving disease control (72). Propolis has been studied for its anticancer and neuroprotective properties. Brazilian green propolis, in particular, has shown a potential to improve cognitive functions and protect against neurodegenerative damage due to its antioxidant properties (73). Propolis has been explored for its potential against SARS-CoV-2 infection mechanisms. It inhibits key pathways involved in viral entry and inflammation, suggesting its utility in managing COVID-19 and related respiratory conditions (74).

Propolis demonstrates complementary effects across different demographics and metabolic conditions. Its benefits vary by gender, with males potentially experiencing more pronounced effects (75). In older adults, propolis aids in reducing body fat and oxidative stress (55, 76), while in metabolic disorders like PCOS and diabetes, it improves insulin sensitivity and lipid profiles. Overall, propolis supports metabolic regulation through its impact on gut microbiota, adipogenesis, and inflammation, making it a versatile nutraceutical for metabolic health (49, 77).

The current study is a comprehensive review of the effect of propolis on inflammatory factors and oxidative stress, which has been examined by a larger number of RCTs than in previous reviews. Also, dose-response analysis has determined the optimal dose and duration of propolis consumption to reduce inflammation and oxidative stress.

However, the present meta-analysis has some limitations that should be mentioned: based on the GRADE of most of the obtained results, they are weak to moderate, so it is still not possible to draw a definite conclusion about the effectiveness of propolis in reducing oxidative stress and inflammation. Another limitation of this study is the result of sensitivity analysis for MCP-1, GPx, and SOD variables, which is associated with uncertainty in the conclusions. On the other hand, different types of propolis have been used in RCTs, which have different flavonoid compounds depending on their geographical location and other factors, and as a result, their effectiveness will be different. Also, the heterogeneity of the intervention population in different RCTs is another limitation of this study. The standardization of propolis formulations is challenged by chemical variability, diverse extraction methods, and complex correlations between chemical composition and biological activity. These limitations impact the reproducibility and comparability of study outcomes, highlighting the need for standardized criteria and methodologies to enhance the reliability of propolis-based research and applications. Future research on propolis should prioritize standardization, clinical efficacy, and understanding its mechanisms of action. Additionally, exploring its potential in aging and neurological health, alongside improving production methods, will significantly advance our knowledge and application of propolis in medicine.

## 5 Conclusion

In conclusion, propolis exerts its effects on reducing oxidative stress and inflammation by reducing CRP, IL-6, TNF-α, and MCP-1 and enhancing TAC, GSH, and GPx. These findings support the traditional use of propolis in treating various diseases related to oxidative stress and inflammation. More RCTs are needed to draw definitive conclusions about the best dose and duration of intervention.

## Data Availability

The original contributions presented in the study are included in the article/Supplementary material, further inquiries can be directed to the corresponding authors.
