# Supplementary material for: Propolis supplementation on inflammatory and oxidative stress biomarkers in adults: a systematic review and meta-analysis of randomized controlled trials
Source: Front Nutr. 2025 May 12;12:1542184. doi: 10.3389/fnut.2025.1542184 (PMC12104767; doi:10.3389/fnut.2025.1542184)
Supplement: Supplementary file 1 [file Table_1.docx]

**Supplementary Material**

A)

B)

C)

D)

E)

F)

**Supplementary Figure 1.** Random-effects meta-regression plots of the association between mean changes in A) CRP; B) IL-6; C) TNF-a; D) MDA; E) TAC; and F) SOD and Propolis dose.

A)

B)

C)

D)

E)

F)

**Supplementary Figure 2.** Random-effects meta-regression plots of the association between mean changes in A) CRP; B) IL-6; C) TNF-a; D) MDA; E) TAC; and F) SOD and intervention duration.

A)

B)

C)

D)

E)

F)

G)

H)

I)

J)

K)

L)

**Supplementary Figure 3.** Dose-response relations between Propolis dosage (mg/day) and duration (week) of Propolis supplementation and mean difference in CRP (A, B), IL-6 (C, D), TNF-a (E, F), MDA (G, H), TAC (I, J), and SOD (K, L).

A)

B)

C)

D)

E)

F)

G)

H)

I)

J)

K)

L)

M)

**Supplementary Figure 4.** Funnel plots for the effect of Propolis intake on A) CRP (mg/L); B) IL-6 (pg/ml); C) TNF-α (pg/ml); D) IL-10 (pg/ml); E) IL-2 (pg/ml); F) IL-8 (pg/ml); G) MCP-1 (pg/ml); H) MDA (nmol/ml); I) TAC (mmol/L); J) GSH (µmol/L); K) GPx (U/L); L) SOD (U/ml); and M) PAB.
